# A Comparison of Different Slicing Planes in Preservation of Major Hippocampal Pathway Fibers in the Mouse

**DOI:** 10.3389/fnana.2017.00107

**Published:** 2017-11-21

**Authors:** Guoxiang Xiong, Hannah Metheny, Brian N. Johnson, Akiva S. Cohen

**Affiliations:** ^1^Department of Anesthesiology and Critical Care Medicine, Children’s Hospital of Philadelphia, Philadelphia, PA, United States; ^2^Department of Anesthesiology and Critical Care Medicine, Perelman School of Medicine, University of Pennsylvania, Philadelphia, PA, United States

**Keywords:** cornu ammonis, trisynaptic circuit, entorhinal input, subiculum, pyramidal cell, alveus

## Abstract

The hippocampus plays a critical role in learning and memory and higher cognitive functions, and its dysfunction has been implicated in various neuropathological disorders. Electrophysiological recording undertaken in live brain slices is one of the most powerful tools for investigating hippocampal cellular and network activities. The plane for cutting the slices determines which afferent and/or efferent connections are best preserved, and there are three commonly used slices: hippocampal-entorhinal cortex (HEC), coronal and transverse. All three slices have been widely used for studying the major afferent hippocampal pathways including the perforant path (PP), the mossy fibers (MFs) and the Schaffer collaterals (SCs). Surprisingly, there has never been a systematic investigation of the anatomical and functional consequences of slicing at a particular angle. In the present study, we focused on how well fiber pathways are preserved from the entorhinal cortex (EC) to the hippocampus, and within the hippocampus, in slices generated by sectioning at different angles. The postmortem neural tract tracer 1,1′-dioctadecyl-3,3,3′3′-tetramethylindocarbocyanine perchlorate (DiI) was used to label afferent fibers to hippocampal principal neurons in fixed slices or whole brains. Laser scanning confocal microscopy was adopted for imaging DiI-labeled axons and terminals. We demonstrated that PP fibers were well preserved in HEC slices, MFs in both HEC and transverse slices and SCs in all three types of slices. Correspondingly, field excitatory postsynaptic potentials (fEPSPs) could be consistently evoked in HEC slices when stimulating PP fibers and recorded in stratum lacunosum-moleculare (sl-m) of area CA1, and when stimulating the dentate granule cell layer (gcl) and recording in stratum lucidum (sl) of area CA3. The MF evoked fEPSPs could not be recorded in CA3 from coronal slices. In contrast to our DiI-tracing data demonstrating severely truncated PP fibers in coronal slices, fEPSPs could still be recorded in CA1 sl-m in this plane, suggesting that an additional afferent fiber pathway other than PP might be involved. The present study increases our understanding of which hippocampal pathways are best preserved in the three most common brain slice preparations, and will help investigators determine the appropriate slices to use for physiological studies depending on the subregion of interest.

## Introduction

Mammalian brains are complex three-dimensional structures, but depending on the question being asked and the region being studied, it may not be either necessary or desirable to investigate their entire structure. The hippocampus is one of the most well studied regions in the brain, and plays a critical role in learning and memory as well as additional higher cognitive functions. It has also been implicated in numerous neuropathological disorders including epilepsy, Alzheimer’s and traumatic brain injury (Kandel et al., 2013). The hippocampus is able to accomplish these complex functions by virtue of its specialized and complicated synaptic circuitry including its fiber connections, and these fibers are frequently studied using brain slices. Brain slices represent a useful compromise between a preparation complete enough to reproduce important phenomena, and yet simple enough to be experimentally tractable. The many technical advantages of brain slices include comparatively straightforward field potential and single cell recording, and straightforward chemical and pharmacological access. Slices may also be able to preserve both local and long range circuitry, but doing so requires careful consideration of how the relevant anatomy will be affected by the slicing process. Chief among these considerations is the angle of sectioning through the desired region.

**Figure 1:**
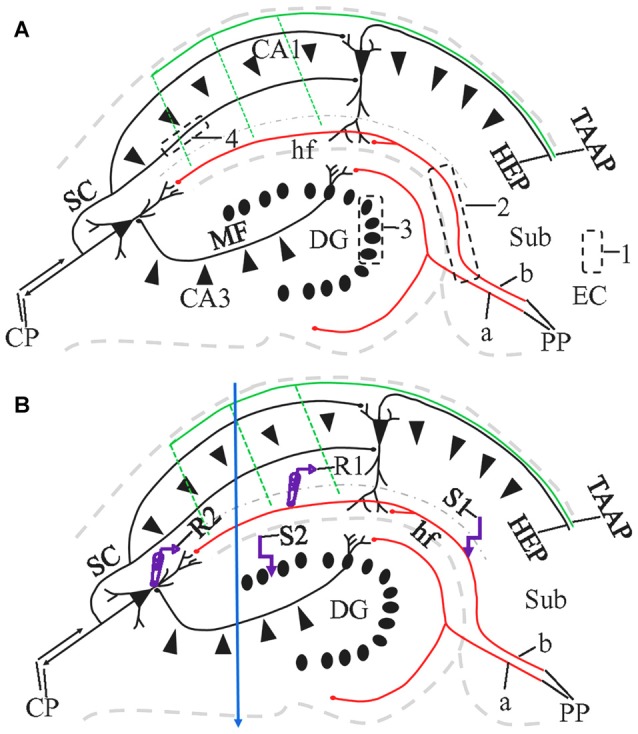
Schematic diagram illustrating the major pathways in the hippocampus, 1,1′-dioctadecyl-3,3,3′3′-tetramethylindocarbocyanineperchlorate (DiI) injection sites and the locations of the stimulating and recording electrodes in a HEC slice. CP, commissural projections; HEP, hippocampal-entorhinal projection; MF, mossy fibers; PP, perforant path (a, PP to Dentate gyrus, DG; b, PP to CA1); SC, Shaffer collaterals; TAAP, temporoammonic alvear pathway. Its branches (dashed parallel lines) may not be well preserved in HEC slices. Dash-dotted curve delineates the boundary of stratum lacunosum-moleculare (sl-m) with stratum radiatum (sr). **(A)** DiI injections illustrated as black dashed rectangles. To trace PP fibers from the cells of origin, DiI was injected into the entorhinal cortex (EC) from fixed slices or whole brains (1). To trace PP fibers from their termination site, we also injected DiI into CA1 and DG, with part of the subiculum (Sub) involved (2). To trace MFs or SCs, DiI injections were made into granule cell layer (gcl; 3) or sr of CA1 (4), respectively. **(B)** Design of field excitatory postsynaptic potential (fEPSP) recording along PP (R1 and S1) or MF pathway (R2 and S2). R, recording electrode; S, stimulating electrode. Blue arrow indicate a razor blade cut extending from the proximal part of CA1 through CA3 to prevent activation of CA1 pyramidal cells from CA3-CA1 or DG-CA3-CA1 projections, when fEPSP was evoked and recorded in sl-m.

The angle of sectioning is particularly relevant to the preservation of long fiber pathways. Among the fiber connections to principal neurons of the hippocampus, the “trisynaptic circuit” (Amaral and Lavenex, 2007) has been most frequently investigated. As classically defined, the trisynaptic circuit (Figure 1) consists of the perforant path (PP) connection from the entorhinal cortex (EC) to dentate gyrus (DG); the mossy fiber (MF) projection from DG to hippocampal area CA3, and the Schaffer collateral (SC) projection from CA3 to hippocampal area CA1. In addition to this classic trisynaptic circuit, there is also a PP projection to stratum lacunosum-moleculare (sl-m), referred to by some investigators as the temporoammonic pathway (TA; see also the “Discussion” section regarding this terminology), although defined by Ramon y Cajal (1911) as part of the PP. Last, and rarely studied, the hippocampus also receives afferent entorhinal input through the temporoammonic alvear pathway (TAAP; Amaral and Lavenex, 2007; Figure 1, green), also known as entorhinal-hippocampal alvear pathway (Ramon y Cajal, 1911) or alvear pathway (Deller et al., 1996).

A more detailed investigation into these connections demonstrates that PP fibers terminate in two different patterns: onto the distal dendrites of the granule cells in the molecular layer (ml) of DG, and also onto the distal dendrites of the pyramidal neurons in CA1-CA3 and the subiculum (Ramon y Cajal, 1911; Steward and Scoville, 1976; van Groen et al., 2002; Witter, 2007). In addition to PP projection, the hippocampus also receives EC input via the less well-studied temporoammonic alvear pathway (Ramon y Cajal, 1911; Deller et al., 1996; Amaral and Lavenex, 2007). This temporoammonic alvear pathway also targets CA1 apical dendrites in sl-m, but does so in a strikingly different manner from the PP sl-m projections; temporoammonic alvear pathway course through the alveus and then make a sharp downward turn toward CA1 pyramidal cell body layer, which they pass through before reaching and branching out in, sl-m (Deller et al., 1996; Amaral and Lavenex, 2007).

Continuing along the trisynaptic circuit, MFs are the axons from dentate granule cells and synapse onto the proximal dendrites of CA3 pyramidal cells in stratum lucidum (sl; Amaral and Lavenex, 2007). SCs are CA3 pyramidal cell axon fibers which terminate onto CA1 pyramidal neurons at proximal apical dendrites in stratum radiatum (sr) and basal dendrites in stratum oriens (Lorente de No, 1934; Swanson et al., 1978; Ishizuka et al., 1990).

There are three common slicing angles that are employed to investigate hippocampal circuitry: transverse, coronal and hippocampal-entorhinal cortex (HEC; described below). Historically, the first live slices prepared from the hippocampus were transverse. These were created by first dissecting out the hippocampus and then slicing the hippocampus transversely along its long axis with surgical blades (Skrede and Westgaard, 1971). Subsequent technical refinements for cutting transverse slices included the use of a tissue chopper (Schwartzkroin, 1975) or vibratome (Andersen, 1977; Dingledine et al., 1977). Physiologists next began to cut coronal slices (also referred to as frontal slices by some researchers) with a vibratome (Otis et al., 1991; Staley et al., 1992), in which the brain is blocked for cutting so that the blade will pass through the hippocampus in the coronal plane. Last, to include as much of EC as possible, and to keep as many intact entorhinal-hippocampal and/or hippocampal-entorhinal fibers as possible, a modified horizontal slice preparation termed the HEC slice was developed, and initially generated using a tissue chopper (Walther et al., 1986; Stanton et al., 1987; Jones and Heinemann, 1988). HEC slices can now be very precisely and reproducibly prepared with a vibratome (Rafiq et al., 1993), by blocking the brain such that the blade effectively passes through at a 12° downward angle from rostral to caudal. This acute angle is also referred as “magic cut” (Bischofberger et al., 2006).

All three slicing preparations have been widely used for investigating hippocampal circuitry (e.g., for investigations of PP in HEC slices: Stepan et al., 2012; Coronal: Yu et al., 2010; Transverse: Bonnici and Kapfhammer, 2009. For MFs in HEC slices: Skucas et al., 2013; Coronal: Kabakov et al., 2012; Transverse: Wiera et al., 2013. For SCs, HEC: Farmer et al., 2013; Coronal: Bagley and Westbrook, 2012; Transverse: Bagley and Westbrook, 2012). The choice of slicing plane generally follows two broad patterns: a significant number of intrahippocampal electrophysiological studies were carried out using transverse slices, while many studies focusing on entorhinal-hippocampal connections were done using HEC slices. Laboratories are likely to prepare brain slices for physiological experiments based on their traditional preference and/or existing equipment. However, the preservation of the major afferent fiber systems within the hippocampus is likely to vary between slicing planes, and heretofore this variation has not been systematically evaluated. For instance, despite the large number of studies using hippocampal brain slices, and the commonly accepted notion that HEC slices are the best choice for studies of interaction between the hippocampus and EC, there have been no direct, anatomical studies of the extent to which the different slicing methods preserve this connection.

In the present study we focused on intrahippocampal fiber preservation in slices generated by sectioning through various planes. The post mortem neural tract tracer 1,1′-dioctadecyl-3,3,3′3′-tetramethylindocarbocyanine perchlorate (DiI) was used to label afferent fibers synapsing onto dendritic arbors of principal neuronal groups by injecting the tracer into either specific hippocampal subregions of fixed slices or into EC in intact fixed whole brains. DiI is a lipophilic dye and passively diffuses through cell membranes. Laser scanning confocal microscopy was adopted for imaging DiI-labeled pathway fibers and their terminals. Our DiI-tracing data demonstrated that all three pathway fibers were well preserved in HEC slices, MFs and SCs were preserved in transverse slices, and only SCs were well preserved in coronal slices. We also investigated the functional consequences of a subset of these anatomical findings by comparing evoked field excitatory postsynaptic potentials (fEPSP) along PP or MF pathways in both HEC and coronal slices. The anatomical evidence presented in this report can help investigators determine the most appropriate slice plane to use for physiological experimentation.

## Materials and Methods

### Tissue Preparation for DiI Tracing

A total of 37 male C57/B6 mice (Jackson Laboratory, Bar Harbor, ME, USA) at 8 weeks of age were used for DiI tracing portion of this study. Mice were kept under a 12 h light/12 h dark cycle, with *ad libitum* access to food and water. The present procedures and protocols for all animal studies were approved by the Children’s Hospital of Philadelphia and UPENN Institutional Animal Care and Use Committees in accordance with international guidelines on the ethical use of animals (National Research Council, 1996). Mice were deeply anesthetized with 5% chloral hydrate and then perfused with saline followed by 4% paraformaldehyde in 0.1 M phosphate buffer (PBS, pH 7.4). Brains were removed and post-fixed in the same fixative for 90 min. They were stored in PBS at 4°C before DiI injections. Perfusion and post-fixation were done at room temperature.

### DiI Tracing in Fixed Slices

HEC, coronal or transverse slices were prepared at 250 μm thickness with a VT 1000S vibratome (Leica, Buffalo Grove, IL, USA). The 250 μm of thickness was determined based on a compromised balance between different techniques used in the present study, i.e., slices of 350 μm or thicker are more appropriate for physiological recording or neural tract tracing, whereas thinner slices are better for confocal microscopy. For HEC slices the blade was advanced in a horizontal plane after elevating the posterior portion of the brain 12° by placing it on a wedge-shaped agar ramp, as detailed by Rafiq et al. (1993). For transverse slices, the hippocampus was first dissected out of the intact brain and then embedded in 5% agarose. The agarose-embedded hippocampus was then cut perpendicular to its long axis. To trace the three major afferent fiber pathways (PP, MF and SC) in the hippocampus, 2% DiI (Invitrogen-Molecular Probes, Grand Island, NY, USA) in N,N-dimethylformamide (Sigma, St. Louis, MO, USA) was injected into each slice (Figure 1A). DiI injections were made with a glass micropipette (10 μm in tip diameter), which was attached to an IM-300 Microinjector (Narishige, East Meadow, NY, USA), as reported previously (Xiong et al., 2007). To label local PP fibers, DiI was injected into sl-m of CA1. In these slices, the dye often diffused into the ml of DG because these layers lie adjacent to each other. To trace MFs, DiI was injected into granule cell layer (gcl) in the various slices. In these slices, careful efforts were made to prevent the dye from directly diffusing into the hilus. We also injected DiI into sr of CA1 to label SCs. In some coronal slices at the caudal level and HEC slices to which EC is attached, DiI was also injected into EC in order to trace PP fibers from EC to the hippocampus. DiI-injected slices were kept in the dark at 37°C for 1 week.

### Tracing PP Fibers in Fixed Whole Brains

Fixed whole brains with intact pia mater were kept in 35 mm petri dish. To trace PP fibers from EC to the hippocampus (Figure 1A), DiI solution was injected into EC using a microinjector with a glass micropipette. To localize layers 2/3 of EC which are the cells of origin of entorhinal-hippocampal projections (Witter et al., 1988; van Groen et al., 2003; Witter, 2007), the micropipette was inserted 200–250 μm deep from the pial surface. The injections were made in multiple sites (0.5 μl at each site), forming three parallel longitudinal rows starting along the line just ventral to the rhinal fissure, with four injection sites running from the rostral to caudal along each row. Injected brains were kept at 37°C for 3 weeks to allow DiI diffusing along cell membranes to the hippocampal subregions. HEC, coronal or transverse slices were cut at 150 μm in thickness from different brains with the VT1000S vibratome.

### Counter Staining, Slice Mounting, Coverslipping and Confocal Imaging

DiI-injected slices and slices cut from DiI-injected whole brains were incubated with Hoechst (a nuclear dye commercially available from Invitrogen-Molecular Probes) at room temperature for 90 min. The slices were then mounted on glass slides and coverslipped with aqueous mounting medium. Samples were stored in the dark at 4°C before observation and imaging with fluorescent microscopes.

Confocal images were acquired with Olympus Fluoview 1000 System. The Z-step size was set at 0.5 μm. For injection sites or overall distribution pattern of DiI-labeled fibers, a 4×, 10× or 20× objective was used. In order to examine detailed structures of labeled fibers, a 40× dry objective was used. A stack of confocal images was projected to show a general distribution pattern, whereas the high power structure of labeled fibers was depicted with single-step images. For each tracing setting, the distribution pattern of DiI-labeled fibers was confirmed in at least three mice. If negative data were acquired as for MF tracing in coronal slices, the experiment was then repeated in one or two extra mice. From each mouse, a minimum of five hippocampal slices were examined for DiI labeling.

### Electrophysiological Recording

Sixteen naïve mice (8 weeks of age) were used for recording fEPSPs evoked by stimulation of the afferent PP and MF pathways. Mice were anesthetized with Isoflurane (Baxter, Deerfield, IL, USA) and decapitated. Brains were quickly and carefully dissected and chilled in oxygenated ice-cold sucrose based cutting medium containing (in mM) 200 Sucrose, 50 NaHCO_3_, 10 Glucose, 2.5 NaH_2_PO_4_, 1 MgCl_2_ and 2 CaCl_2_. Either HEC or coronal slices at 350 μm thickness were prepared from each brain with a VT 1200S vibratome (Leica). Live slices were maintained in oxygenated artificial cerebral spinal fluid (aCSF) containing (in mM) 130 NaCl, 3 KCl, 1.25 NaH_2_PO_4_, 25 NaHCO_3_, 10 Glucose, 1 MgCl_2_ and 2 CaCl_2_, and kept in a 34–35° water-bath for at least 1 h before being transferred to the recording chamber. One brain typically yields 3–4 HEC slices, from which the hippocampal circuitry (including DG, CA1 and CA3) can be clearly identified under a dissecting microscope.

Field EPSPs evoked by PP stimulation were recorded in sl-m of CA1 (Figure 1B) with an Axopatch 1D amplifier (Axon Instruments, Union City, CA, USA) and the Clampex 9.2 data acquisition program (Molecular Devices, Sunnyvale, CA, USA), as previously reported (Schwarzbach et al., 2006; Johnson et al., 2014). Stimulation was applied at the distal end of sl-m via a concentric and bipolar tungsten electrode (Frederick Haer Corporation, Bowdoin, ME, USA). The recording electrode for evoked field potentials were pulled from borosilicate glass (World Precision Instruments, Sarasota, FL, USA) with a tip resistance of 2–6 MΩ when filled with aCSF. The recording electrode was also placed in sl-m, proximal to the stimulation electrode such that orthodromic responses were produced at the recording electrode. Both electrodes targeted the median one-third of sl-m dorsoventrally, with a minimal distance of 600 μm between the two electrodes. A razor blade cut (Figure 1B, blue arrow) was made at the proximal point of CA1 to prevent activation of CA pyramidal cells disynaptically from area CA3 or trisynaptically from DG and CA3, as performed by other groups (Colbert and Levy, 1992; Empson and Heinemann, 1995). For CA3 field recording (Figure 1B), the stimulating electrode was placed in the lateral part of the suprapyramidal blade of gcl. The recording electrode was located at sl of subregion CA3a/b, with at least 600 μm between recording and stimulating electrodes. All extracellular recording experiments were performed at room temperature, in an interface chamber (Scientific Systems Inc., State College, PA, USA) with an aCSF flow rate of 2.0 ml/min. Field potentials were recorded with single stimuli (100 μs in duration) ranging from 50 μA to 1000 μA in 50 μA increments to generate input/output (I/O) curves. The inter-stimulus interval for these field potentials was 8 s. Responses were quantified as the slope of the early, pseudo-linear portion of the response, and comparisons were made at the stimulus strength which gave an approximately half-maximal response, as determined by the I/O curve recorded in each slice. To check for release probabilies, 10 pairs of stimuli were also delivered, with a paired pulse inter-stimulus interval of 75 ms. From each animal, 2–3 slices were recorded.

To identify different components of the response in the recordings, chemical reagents were sequentially applied to the slices as needed. (2R)-amino-5-phosphonovaleric acid (APV, 50 μM; Abcam, Cambridge, MA, USA) together with 6-cyano-7-nitroquinoxaline-2,3-dione (CNQX, 6 μM; Abcam) were used to block excitatory responses in the PP. To block the inhibitory component of the responses, bicuculline methiodide (BMI, 30 μM; Abcam) was applied. To selectively block MF-pyramidal cell transmission (Uchigashima et al., 2007), (1R, 2R)-3-[(1S)-1-amino-2-hydroxy-2-oxoethyl] cyclopropane-1,2-dicarboxylic acid (DCG-IV, 2 μM; Tocris Bioscience, Avonmouth, Bristol, BS11 9QD United Kingdom), a group II-specific agonist for metabotropic glutamate receptors was added to the superfusing aCSF. To isolate stimulation and/or system artifacts, we applied tetrodotoxin (TTX, a sodium channel blocker; 0.4 μM; Abcam) to block all biological responses.

For comparison of the evoked fEPSPs between HEC and coronal slices, we included only the stable baseline periods of the recordings, i.e., responses collected during repeated stimulation at an inter-stimulus interval of 30 s, and after the response to the bath applied reagents had reached a steady value. All of the traces acquired during the stable baseline period for each recording in a given condition were averaged together, and shown as a single waveform with Clampfit 9.2 program (Molecular Devices, Sunnyvale, CA, USA). The waveforms of all conditions were merged together to show differences in an identical slice.

Field potentials were analyzed by measuring the slope of the EPSP over the linear region of the initial portion of the response, and using stable responses acquired during the last 5 min (10 traces) of recording for a given condition. For each slice, comparisons were done using 10 individual slope measurements for a given slice under the different measurement conditions. For group analysis, the responses for each slice in the control condition and in the test condition, were normalized by the average value for each slice in the control condition. A one-way analysis of variance (ANOVA) with Bonferroni Multiple Comparison Test was conducted for the data from recording in CA1 sl-m. For the MF two-group analysis, a Student’s *t* test was performed. *P* values shown in the text were generated from group comparisons. EPSP slopes shown as Mean ± SEM.

## Results

### PP Fibers Traced after DiI Injection into Fixed Slices

**Figure 2:**
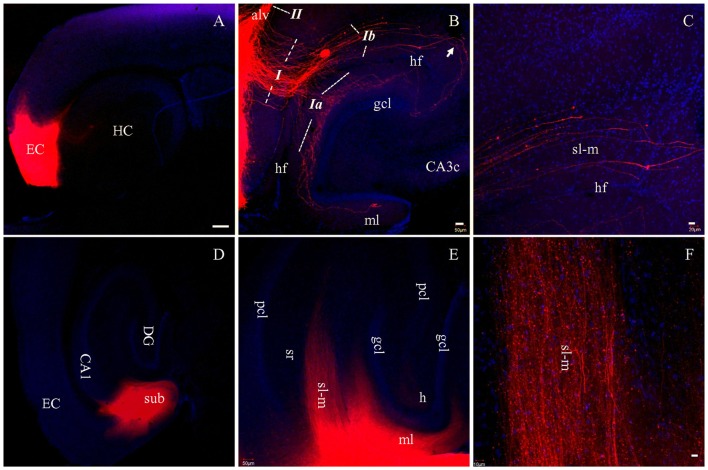
PP fibers labeled by DiI injected into EC **(A–C)** or CA1 and subiculum **(D–F)** in fixed HEC slices. **(A)** DiI injection site (red) in the EC. **(B)** DiI-labeled PP fibers along both banks of the hippocampal fissure (hf). Italicized Arabic numerals with dashed lines indicate entorhinal-hippocampal pathways: *I*, PP fibers coursing toward DG and cornu ammonis (CA); *Ia*, PP fibers to the DG; *Ib*, PP fibers to area CA1-3 subregions. *II*, Temporoammonic alvear path fibers entering the alveus (alv). PP fibers in sl-m are visible continuously from CA1 to CA3 (transition portion indicated by *Arrow*). **(C)** Higher magnification of PP fibers in sl-m of area CA1. PP fibers in CA1 are restricted to sl-m as expected, and can be easily followed. **(D)** DiI injection site in CA1 and subiculum (sub), in a HEC slice. **(E)** General distribution pattern of DiI-labeled fibers in sl-m of CA1 and molecular layer (ml) of DG. **(F)** Higher magnification of DiI-labeled fibers in sl-m of CA1. The slices were counterstained with Hoechst, a nuclear dye (blue). CA3c, subregion c of are CA3; gcl, granule cell layer of DG; h, hilus of DG; HC, hippocampus; pcl, pyramidal cell layer of CA1-3; sr, stratum radiatum. Scale bars: 250 μm in **(A,D)**, 50 μm in **(B,E)**, 20 μm in **(C)** and 10 μm in **(F)**.

To determine how well PP fibers were preserved by the plane of sectioning during the preparation of live brain slices, we prepared fixed slices for DiI tracing. After DiI injections into EC in HEC slices (Figure 2A, red), a large bundle of labeled fibers were seen perforating the subiculum upon entry into the hippocampus (Figure 2B, *I*). This fiber bundle (*I*) perfectly follows the route of PP, as described previously (Ramon y Cajal, 1911; Amaral and Lavenex, 2007). This bundle could be further divided into two groups: the first group crossed the hippocampal fissure (hf) and was distributed in the outer portion of ml of DG (Figure 2B, *Ia*); whereas, the second group entered sl-m of CA1 and traveled all the way to its proximal pole (Figures 2B,C, *Ib*). These large sized fibers traveling along sl-m (*Ib*) could be clearly seen traversing CA1 to reach CA3 (Figure 2B, *arrow*) and terminating at a white mater point adjacent to the tip of the suprapyramidal blade of gcl. We also identified another large bundle of DiI-labeled fibers (*II*) entering and coursing along the alveus (Figure 2B), which has been referred to as the temporoammonic alvear pathway (Ramon y Cajal, 1911; Deller et al., 1996; Amaral and Lavenex, 2007). However, these fibers could not be followed to their terminal destination in sl-m in HEC slices.

In order to trace PP fibers from terminal sites within the hippocampus, we also injected DiI directly into CA1 and DG in HEC slices. Figure 2D shows DiI injection centered at CA1, with part of the subiculum involved. Densely labeled fibers were arranged in parallel with each other in sl-m (Figures 2E,F) and ml (Figure 2E). In CA1, DiI-labeled large diameter fibers could be clearly traced through sl-m, from the boundary of the injection site all the way to their fine tips (Figure 2F). In DG, ml was rich in DiI labeled axon collaterals and terminals (Figure 2E).

**Figure 3:**
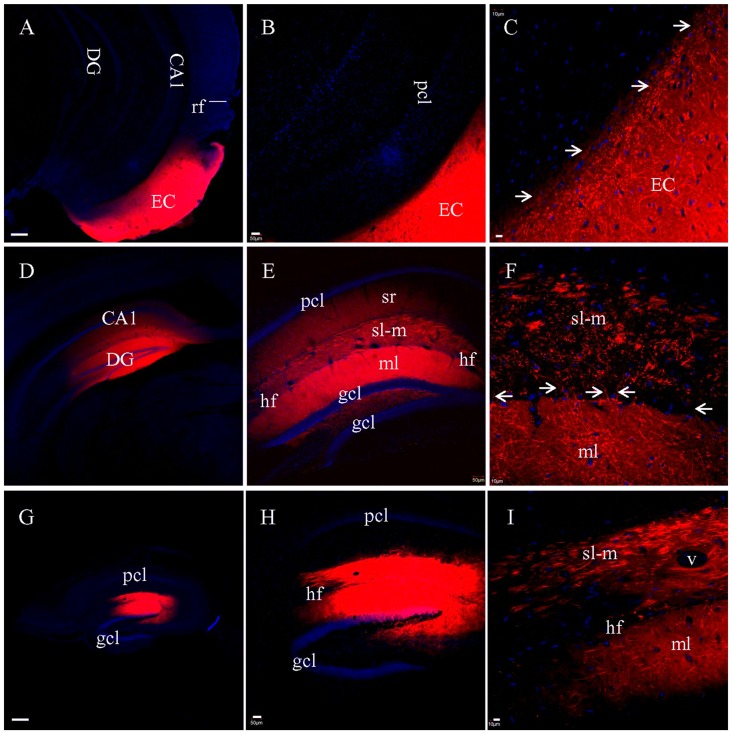
PP fibers labeled by DiI injected into fixed coronal **(A–F)** or transverse **(G–I)** slices. **(A)** DiI injection site in EC from a coronal slice. rf, rhinal fissure. **(B)** No labeled fibers could be seen passing from EC into the hippocampus. **(C)** Higher magnification demonstrating that DiI-labeled fibers were truncated along the medial edge of EC (*arrows*). **(D)** DiI injections in CA1 and DG from a coronal slice. **(E)** General distribution of DiI-labeling. **(F)** Higher magnification showing truncated fibers labeled in sl-m of CA1. Some DiI-labeled fibers (*arrows*) could be clearly tracked, crossing the hf to enter ml and running in distances therein. **(G)** DiI injections in CA1 and DG from a transverse slice. **(H)** General distribution of DiI-labeling. **(I)** Higher magnification showing that DiI-labeled fibers in both sl-m and ml were segmented. Scale bars: 250 μm in **(A,D,G)**, 50 μm in **(B,E,H)**, and 10 μm in **(C,F,I)**.

When DiI was injected into EC (Figure 3A) in coronal slices at the caudal-most level, where slices transect both EC and hippocampus, no DiI-labeled fibers could be tracked into the hippocampus (Figures 3B,C). Interestingly, numerous fiber truncations could be identified along the medial edge of EC (Figure 3C, *arrows*). After DiI injections into CA1 and DG in coronal slices (Figure 3D), both sl-m and ml were intensely labeled (Figure 3E), although no DiI-labeled fibers could be followed for any significant length along sl-m (Figure 3E). Labeled PP fibers in sl-m were uniformly truncated into clusters of short segments (Figure 3F), except for a few labeled fibers traversing the hf (Figure 3F, *arrows*). Within ml of DG, numerous labeled fibers were observed coursing in various directions and length (Figure 3F). In transverse slices where EC is removed during slice preparation, only termination site tracing is possible. DiI injections into CA1 and DG (Figure 3G) resulted in segmented fiber bundles (Figures 3H,I). These results from DiI tracing in fixed slices indicate that PP fibers are well preserved in HEC slices, but segmented in transverse slices and severely truncated in coronal slices.

### Entorhinal-Hippocampal Projection Fibers Traced after DiI Injection into Fixed Whole Brains

**Figure 4:**
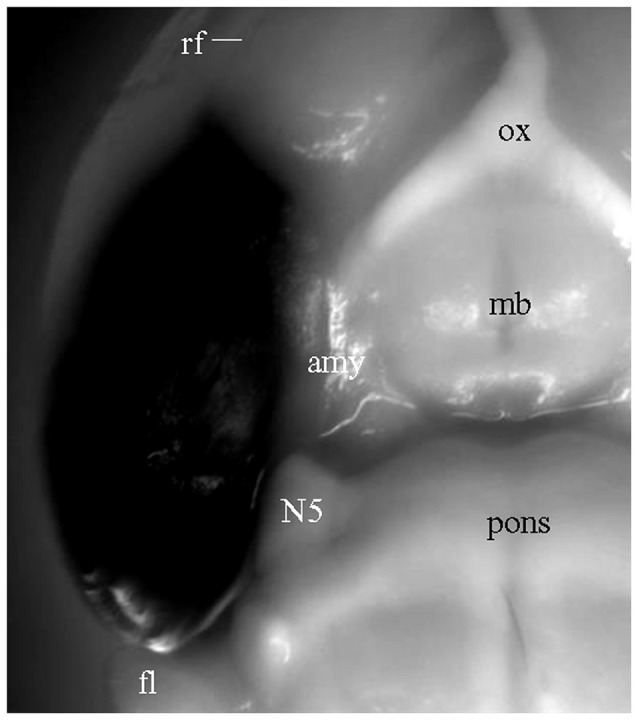
Ventrolateral view of a fixed whole brain with DiI injections covering the entire surface of EC. Part of perirhinal and piriform cortices may also have been involved. Converted grayscale photograph showing DiI as black. amy, amygdala; fl, flocculus of the cerebellum; mb, mammillary body; N5, trigeminal nerve; ox, optic chiasm.

**Figure 5:**
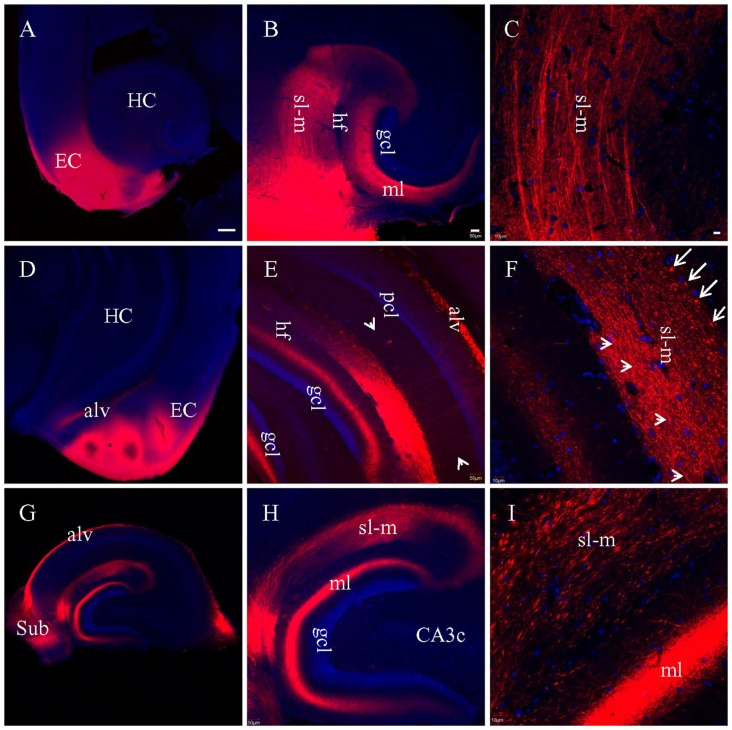
Labeled entorhinal-hippocampal fibers in HEC **(A–C)**, coronal **(D–F)** and transverse **(G–I)** slices 3 weeks after DiI injections into EC of fixed whole brains. **(A)** DiI injections shown in a HEC slice. **(B)** General distribution pattern of DiI labeling in the hippocampus from the same slice as in **(A)**. **(C)** Higher magnification demonstrating DiI-labeled PP fiber bundles in sl-m. **(D)** DiI injections in a coronal slice. **(E)** General distribution pattern of DiI labeling in the hippocampus from a slice slightly rostral to **(D)**. *Arrowheads* indicate temporoammonic alvear path fibers. **(F)** Higher magnification showing DiI-labeled axonal collaterals in sl-m of CA1 and ml of DG. Fibers in large diameter were segmented in various length (*arrowheads*) or transversely truncated (*arrows*). **(G)** Overall distribution pattern of DiI labeling in a transverse slice. **(H)** Loop-like band of DiI-labeled fibers from sl-m to ml. **(I)** Higher magnification showing segmented DiI-labeled PP fiber bundles in sl-m of CA1 and high density of DiI-labeled terminals and axonal collaterals in ml. Scale bars: 250 μm in **(A**,**D,G)**; 50 μm in **(B**,**E,H)**; 10 μm in **(C**,**F,I)**.

For the experiments above in which DiI was injected into brain slices, long processes showing DiI labeling will only be visible for cells which took up the dye at the injection site, and which have long, unbroken processes wholly contained within the physical volume of the slice. That is, if the portion of EC present in a brain slice is not anatomically connected to the portion of hippocampus present in that same slice, then DiI injected in EC will not be able to reach the hippocampus via passive diffusion of the dye. It is possible, however, that the absence of long labeled processes noted in slices that showed only truncated fibers was instead a technical limitation of our ability to visualize long labeled processes. To confirm that the absence of long labeled fibers was not simply a technical limitation of our slicing protocols, we injected DiI into EC of fixed whole brains, waited 3 weeks for injected DiI to diffuse to the hippocampal subregions, and then prepared brain slices. Under a surgical microscope, EC was located by the following landmarks: ventral to the rhinal fissure, lateral and caudal to the amygdala, and rostral to the cerebellum (Figure 4). Multiple injections were undertaken in order to inject the entire surface of EC (see “Materials and Methods” section for details). In HEC (Figure 5A) and coronal (Figure 5D) slices both EC and hippocampus are transected, the injection sites of DiI (*arrows*) are clearly visible. There was no evidence of direct (non-membrane mediated) long range DiI diffusion to the hippocampus. Although two intense bands of DiI-labeled PP fibers could be identified in sl-m and ml in both HEC (Figure 5B) and coronal (Figure 5E) slices, only in HEC slices could intact DiI-labeled fibers of large diameter be traced for long distances (Figure 5C). In contrast to HEC slices, coronal slices exhibited numerous DiI-labeled beaded axonal collaterals and terminals (synaptic boutons), without any large diameter long fibers (Figure 5F). Instead, fiber segments of various lengths (Figure 5F, *arrowheads*) or fiber transverse truncations (Figure 5F, *arrows*) could be clearly identified in sl-m. In transverse slices (Figure 5G), DiI-labeling was present in a complete loop-like band from sl-m to ml, even though the labeling in ml was present at much higher intensity (Figure 5H). While DiI-labeled fibers were segmented in sl-m, homogeneous beading of axonal collaterals was prominent in ml (Figure 5I). No CA1 pyramidal cell bodies and/or dendritic trees could be identified in HEC, coronal or transverse slices after DiI injections into EC. These results indicate that the present protocol did not result in detectable retrograde labeling of hippocampo-entorhinal projections.

**Figure 6:**
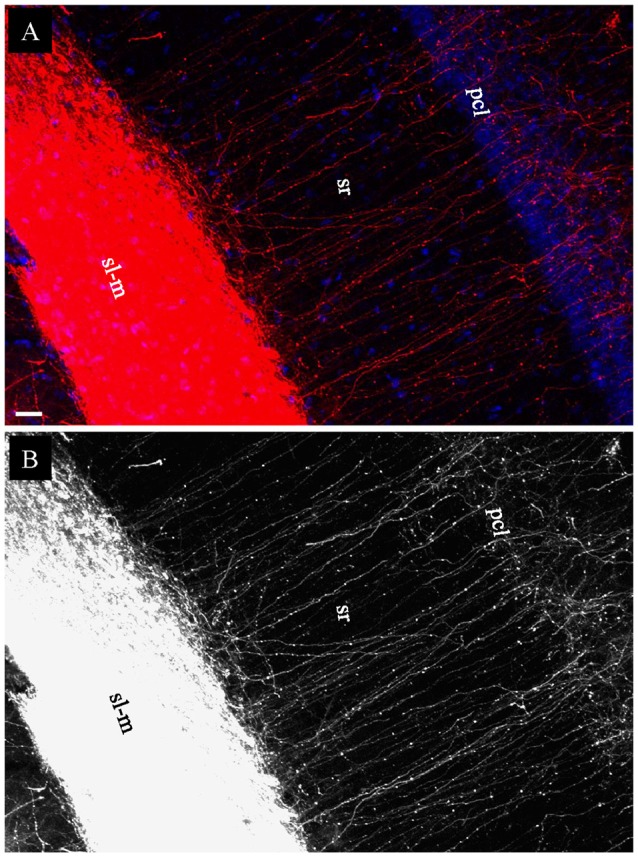
Labeled temporoammonic alvear pathway fibers in coronal slices at the caudal level, 3 weeks after DiI injections into EC of a fixed whole brain. **(A)** Higher magnification of Figure 5E (*arrowheads*) showing DiI-labeled fibers perforating pcl and sr of CA1 before terminating in sl-m. **(B)** Grayscale of **(A)**, highlighting DiI-labeled fibers running parallel in sr. Scale bar: 20 μm.

In coronal slices prepared after EC injection in fixed whole brains, but not in HEC or transverse slices, a large group of DiI-labeled fibers were seen exiting the alveus by making an abrupt right angle turn (Figure 5E, *arrows*). These fibers were then arranged in parallel and perforated the pyramidal cell layer (pcl) and sr before branching and terminating in sl-m (Figures 6A,B). This striking anatomy has been described previously (Deller et al., 1996) and these laminae crossing fibers has been identified as temporoammonic alvear pathway (Deller et al., 1996; Amaral and Lavenex, 2007), although the function of these connections is unknown.

### MFs Traced in Fixed Slices

**Figure 7:**
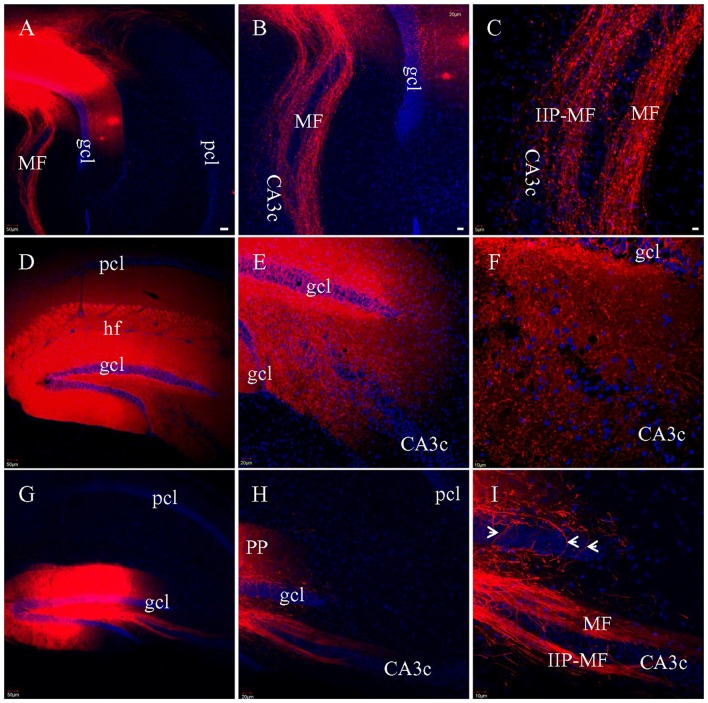
Labeled MFs after multiple injections of DiI into gcl in fixed slices. **(A)** DiI injections and labeled MFs in a HEC slice. **(B)** DiI-labeled MF bundles in the same slice as in **(A)**. **(C)** Higher magnification of **(B)**, showing DiI-labeled MFs and presynaptic terminals (boutons). IIP-MF: Intra-/infrapyramidal MF projection. **(D)** Broad DiI injections centered at DG in a coronal slice. **(E)** No long, intact MF bundles labeled, only diffuse and punctate labeling. **(F)** Higher magnification of **(E)**. DiI-labeled terminals mixed with truncated fibers. **(G)** DiI injections and labeled MFs in a transverse slice. **(H)** DiI-labeled MF bundles. **(I)** Higher magnification of **(H)** showing two bundles of DiI-labeled MFs, with some of them segmented. Axons from hilar mossy cells might be also labeled *(arrowheads*). Scale bars: 50 μm in **(A**,**D,G)**; 20 μm in **(B**,**E,H)**; 10 μm in **(C**,**F,I)**.

To assess MF preservation in the various slicing protocols, multiple injections of DiI was made directly into gcl in fixed HEC (Figure 7A), coronal or transverse slices (Figure 7G). In both HEC (Figures 7B,C) and transverse (Figures 7H,I) slices, two bundles of labeled MFs could be clearly identified as classical and intra-/infrapyramidal MF projections (Swanson et al., 1978; Lipp et al., 1987), coursing from the hilus to CA3 (Figures 7B,H). In HEC slices, individual intact MFs could be traced for long distances among MF terminals (Figure 7C). In transverse slices, a subset of the MFs were truncated into segments of shorter and variable lengths (Figure 7I). Unlike HEC slices, transverse slices appeared not to exhibit axonal collaterals and the MF terminals. In contrast, DiI injections into gcl in coronal slices could not successfully label either bundle of MFs. We then made broad DiI injections in other coronal slices such that both gcl and the hilus were covered (Figure 7D). However, from these later slices, we still could not identify long DiI-labeled MFs (Figures 7E,F). Instead, we could only see punctate labeling in the hilus, suggestive of direct DiI diffusion (not the passive diffusion via cell membranes) to large-sized MF terminals (Figure 7F). It is hard to tell if these punctate labeling includes transversely truncated MF axons.

### SCs Labeled in Fixed Slices

**Figure 8:**
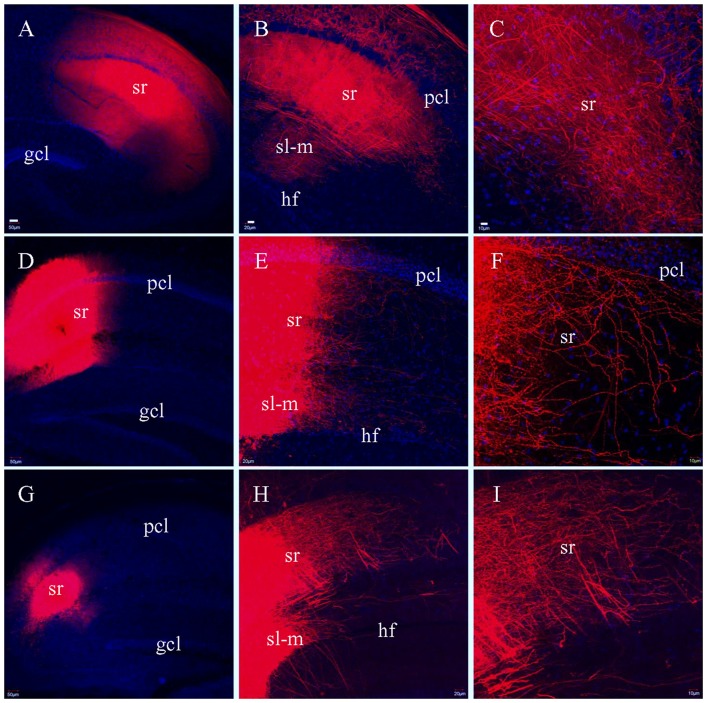
Labeled fibers in sr of CA1 after DiI was injected into the same layer in fixed slices. **(A)** DiI injections centered at sr in a HEC slice. **(B)** DiI-labeled fibers in sr from the same slice as in **(A)**. **(C)** Higher magnification of **(B)** showing DiI-labeled fibers. **(D)** DiI injections centered at sr in a coronal slice. **(E)** DiI-labeled fibers in sr and sl-m from the same slice as in **(D)**. **(F)** Higher magnification of **(E)** showing DiI-labeled axonal collaterals bearing presynaptic terminals in sr. **(G)** DiI injections centered at sr in a transverse slice. **(H)** DiI-labeled fibers in sr and sl-m from the same slice as in **(G)**. **(I)** Higher magnification of **(H)** showing DiI-labeled fibers in sr. Scale bars: 50 μm in **(A**,**D,G)**; 20 μm in **(B**,**E,H)**; 10 μm in **(C**,**F,I)**.

To determine if there was variation in how well SCs were preserved by the different sectioning methods, we attempted to label SCs by injecting DiI into CA3 pcl, but this did not result in efficient labeling of SCs in CA1. We then injected DiI into sr of area CA1 in HEC (Figure 8A), coronal (Figure 8D) or transverse slices (Figure 8G), hoping to label SCs at their terminating site. Unlike the PP and MF pathways which demonstrated different fiber distribution patterns in the different slices, DiI injections in sr resulted in similar distributions of labeled SCs in all three types of slices (Figures 8B,E,H). Under high magnification, numerous terminal-bearing axonal collaterals could be clearly identified in (Figures 8C,F,I).

### fEPSP Recording Along PP and MF Pathways

**Figure 9:**
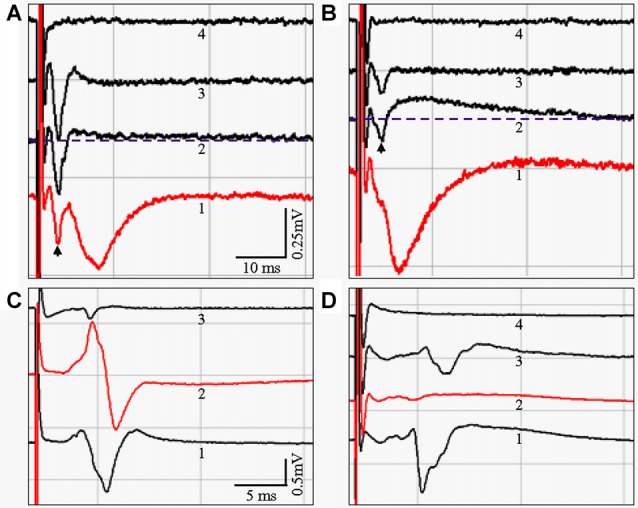
**(A,B)** fEPSP evoked in sl-m of area CA1 from live HEC **(A)** or coronal **(B)** slices, respectively. Waveform shapes and amplitudes were stable after complete wash-in of the indicated recording solutions. Control fEPSPs with a duration over 10 ms were taken from slices bathed in artificial cerebral spinal fluid (aCSF; Waveform 1). In the presence of APV and CNQX, the downward fEPSP signal was blocked (Waveform 2). In addition, a slow, long-lasting upward component appeared, elevating the later portion of the response above the pre-stimulus baseline (*Dashed line*). Application of bicuculline suppressed the APV/CNQX-induced upward signal, leaving only the enlarged fiber volley (Waveform 3). When TTX was added to the three inhibitors, the fiber volley was blocked, and all biological signals disappeared (Waveform 4). **(C,D)** fEPSPs could be evoked in sl of CA3a/b by stimulating gcl from live HEC but not coronal slices. **(C)** A downward signal with a duration over 4 ms could be reliably recorded from HEC slices, and will be referred to here as the MF fEPSP (Waveform C1). When the recording electrode was moved into sr, an upward component appeared (Waveform C2). In coronal slices, only a very small signal with a much shorter duration was recorded (Waveform 3), and only in one of the 12 coronal slices from four mice. Evoking this response also required a much higher stimulation intensity. **(D)** The MF fEPSP (Waveform 1) could be blocked with DCG-IV (Waveform D2). A residual signal with a smaller magnitude and a longer delay could be acquired after DCG-IV was washed away (Waveform 3). The biological signal was completely blocked with the application of TTX, leaving only the stimulus artifact (Waveform 4). Scale bar: 10 ms and 0.25 mV in **(A,B)**; 5 ms and 0.5 mV in **(C,D)**.

To determine if there was a functional correlate to the anatomical differences we observed in PP fiber preservation, we conducted extracellular field recordings in sl-m of CA1 undertaken in live HEC and coronal slices. Figure 9A shows representative fEPSPs evoked and recorded in sl-m of a HEC slice, with the stimulation (200 μA) and recording electrodes placed 1200 μm apart and both in sl-m. A downward fEPSP over 10 ms in duration could be recorded, usually following the fiber volley (waveform 1, *arrow*). In the presence of both APV and CNQX to inhibit NMDA and AMPA receptors respectively, the downward fEPSP signal was blocked (aCSF; one slice per mouse, and *n* = 4 slices/mice): −0.163 ± 0.037 mV/ms vs. APV + CNQX: 0.047 ± 0.042 mV/ms; *P* < 0.05; waveform 2) and the amplitude of the fiber volley seemed to be enlarged (−0.743 ± 0.320 mV/ms) but the difference was not significant when compared to the aCSF group (−0.430 ± 0.224 mV/ms, *P* > 0.05). In addition, a long-lasting upward signal appeared (above the *dotted line*). When bicuculline was added to the inhibitor mixture the long-lasting upright signal was suppressed (fEPSP: 0.004 ± 0.002 mV/ms; *P* < 0.05, compared to aCSF), leaving a further enlarged fiber volley (−0.9765 ± 0.3663 mV/ms; *P* < 0.05, compared to aCSF group; waveform 3). When TTX was added to the above drug containing aCSF, all biological signals were blocked, including the fEPSP (fEPSP 0.005 ± 0.002 mV/ms; *P* < 0.05, compared to aCSF) and the fiber volley (0.009 ± 0.011 mV/ms, *P* < 0.05, compared to other three groups; waveform 4), leaving only the stimulus artifact. Similar fEPSPs could be recorded in sl-m from coronal slices (Figure 9B), at the median to caudal levels from around 2.30–3.40 mm posterior to Bregma that are equivalent to Figures 50–59 in the mouse brain atlas by Paxinos and Franklin (2001). However, the stimulation and recording electrodes were necessarily placed closer together (600–700 μm) in coronal slices than in HEC slices. Furthermore, a higher stimulation intensity (350–500 μA) was needed to evoke fEPSPs from coronal slices compared to HEC slices. A one way ANOVA analysis demonstrated that the fEPSP of the aCSF group (*n* = 5 slices, one slice per mouse, −0.134 ± 0.012 mV/ms) were suppressed by APV + CNQX (−0.007 ± 0.020, *P* < 0.05), APV + CNQX + BMI (0.021 ± 0.027 mV/ms, *P* < 0.05), and APV + CNQX + BMI + TTX (0.008 ± 0.008 mV/ms, *P* < 0.05). Although the fiber volley seemed to be slightly increased after treatment with the inhibitors (APV + CNQX: −0.216 ± 0.113 mV/ms; APV + CNQX + BMI: −0.263 ± 0.193 mV/ms), no statistical difference was found when compared to aCSF (−0.175 ± 0.080 mV/ms; *P* > 0.05). When TTX was added to the above three inhibitors, fiber volley was blocked (0.007 ± 0.014 mV/ms, *P* < 0.05), compared to other three groups. At the rostral level (dorsal hippocampus), we could not evoke and record fEPSPs in sl-m of CA1 in coronal slices.

To see if there were functional differences in how well the MF pathway was preserved in HEC vs. coronal slices, we initially attempted to record fEPSPs in sl of CA3 in response to stimulation of ml of DG. Consistent with previous reports (Urban and Barrionuevo, 1996; Vogt and Nicoll, 1999), we were unable to induce measurable responses with this approach. We therefore stimulated gcl of DG, and recorded fEPSPs in sl and sr of area CA3a/b in both HEC and coronal slices (Figures 9C,D). When the recording electrode was in sl, a downward signal with a duration over 4 ms could be reliably evoked in HEC slices from all three mice tested (Figures 9C,D, waveforms C1 and D1), and was qualitatively similar to MF fEPSPs previously described (Castillo et al., 1994; Urban and Barrionuevo, 1996). When the recording electrode was moved into sr, and with the stimulating electrode still in gcl, an upward signal would appear (Figure 9C, waveform 2). The fEPSP evoked in sl by gcl stimulation (aCSF, *n* = 4 slices/mice, −0.712 ± 0.464 mV/ms) could be blocked with DCG-IV (−0.047 ± 0.074 mV/ms, *P* = 1.81 × 10^−18^ detected by Student’s *T* test; Figure 9D, waveform 2), confirming that it was due to MF-pyramidal cell transmission (Uchigashima et al., 2007). After DCG-IV was washed out, a residual signal could be acquired with a smaller magnitude and a slightly longer delay (Figure 9D, waveform 3). The biological signal could be completely blocked with the application of TTX (Figure 9D, waveform 4). In contrast to the sl responses observed in HEC slices, only a very small sl response was recorded in coronal slices after gcl stimulation. This coronal slice signal had a much shorter duration, occurred only once out of 12 coronal slices from four mice, and only when stronger stimulation (500 μA) was applied (Figure 9C, waveform 3).

## Discussion

### Major Findings of the Present Study

In order to evaluate the impact of various slicing angles on afferent fiber preservation in hippocampal slices, we used neural tract tracing with DiI to label PP, MF and SC fibers, and also recorded specific pathway evoked fEPSPs. PP tracing experiments yielded the following results: fully intact labeled PP fibers in HEC slices, segmented fibers in transverse slices, and severely truncated fibers in coronal slices. These results are consistent with the original description by Ramon y Cajal (1911) which noted that “coronal (frontal) sections present these fibers cut across or obliquely”. A potential confound in our DiI labeling studies is the possible inclusion of local fibers from other neurons such as hippocampal interneurons (Freund and Buzsáki, 1996) that may also be labeled. It has also been documented that sl-m receives prominent input from the thalamic nucleus reuniens (Amaral and Cowan, 1980; Wouterlood et al., 1990; Dolleman-Van Der Weel and Witter, 1996; Amaral and Witter, 2000; Vertes, 2015). In addition, sl-m also receives a fair projection from the amygdala and a lighter density of afferents from the raphe nuclei and locus coeruleus (Krettek and Price, 1977; Pasquier and Reinoso-Suarez, 1978; Amaral and Witter, 2000). To exclude all these long projections, and also local fibers other than PP, we injected DiI into EC of fixed brain slices, attempting to trace fiber connections from their cells of origin. PP fibers could be easily tracked from EC to DG and to CA1-3 in HEC slices, in accordance with the PP classification established by Ramon y Cajal (1911). DiI injection into fixed whole brains was also intended to label as many PP fibers as possible, and these whole brain results confirmed the data acquired from tracing fibers by DiI injection into fixed slices.

In addition to the PP projection from EC to hippocampus, our tracer injections into EC also allowed us to visualize the temporoammonic alvear pathway. These fibers are difficult to trace, and thus have been the subject of limited studies. For instance, due to interference from intrinsic fibers, these alvear pathway fibers could not be clearly traced to their termination sites using the Golgi method (Ramon y Cajal, 1911). Using anterograde tracing with Phaseolus vulgaris leucoagglutinin (PhAL), Deller et al. (1996) revisited the alvear pathway. This staining showed that temporoammonic alvear pathway fibers make a sharp right-angle turn in the alveus, perforate pcl and sr, and then branch and terminate in sl-m of CA1. We verified the paths of these alvear fibers, in coronal slices at median to caudal levels, but not in HEC slices. Deller et al. (1996) however, showed temporoammonic alvear fibers in both coronal (frontal) and horizontal slices. This discrepancy might be due to the slight difference in slicing angle, since our HEC slices are tilted 12° off the horizontal plane (Rafiq et al., 1993; Bischofberger et al., 2006).

Of relevance to the present study, the term temporoammonic pathway (“TA path”) has been widely adopted in physiological studies (Maccaferri and McBain, 1995; Dvorak-Carbone and Schuman, 1999; Ang et al., 2005; Fidzinski et al., 2011; Booth et al., 2016). This term is used to refer to a direct entorhinal projection to CA1 and these fibers are discussed as though they are independent from PP (Dvorak-Carbone and Schuman, 1999). As previously described (Dvorak-Carbone and Schuman, 1999; Ang et al., 2005), these “TA” fibers are present just above hf, coursing along and terminating in sl-m of CA1. After careful comparison, we believe that the “TA path” should actually be considered part of PP fibers that innervate CA1 (Ramon y Cajal, 1911; Amaral and Lavenex, 2007), equivalent to the *Ib* as shown in our Figure 2B.

In addition to the PP fibers, DiI was also used to trace MFs and SCs in fixed slices. We successfully traced both classical and intra-/infrapyramidal MF bundles (Swanson et al., 1978; Lipp et al., 1987) after DiI injections in gcl from HEC and transverse slices. By contrast, in coronal slices MFs were so severely truncated that not a single fiber bundle could be followed. After DiI was injected into sr of CA1, we could label well preserved SC fibers in all three types of slices. We attempted to label SCs via DiI injections into the CA3 pcl, which contains the cells of origin of SCs (Swanson et al., 1978; Ishizuka et al., 1990; Amaral and Lavenex, 2007), but could not get efficient labeling in any of HEC, coronal or transverse slices. We therefore injected the tracer directly into sr of CA1 to label SCs at their termination sites and saw similar labeling with all three types of slices. With the present protocol, we were unable to prevent commissural CA3-CA1 projection fibers from being labeled, since these contralateral side-projecting fibers take the same route in CA1 as ipsilateral SCs (Fricke and Cowan, 1987; Amaral and Lavenex, 2007). As a result, we here refer to all DiI-labeled fibers traveling along sr of CA1 as SCs, without excluding the commissural fibers. It has been reported that the CA1 sr may receive noradrenergic and serotoninergic inputs from the locus coeruleus and raphe nuclei (Pasquier and Reinoso-Suarez, 1978), however, these fibers are more limited in density compared to the density of fibers from those same areas projecting to sl-m (Swanson et al., 1987). Similarly, the deep nuclei of amygdala send a small number of fibers into sr but the majority of these fibers are distributed in sl-m (Krettek and Price, 1977). By contrast, septal nuclei project predominantly to the oriens (Nyakas et al., 1987) although a few of these cholinergic fibers may enter sr of CA1 as well (Swanson et al., 1987). At the present, it is hard to discriminate what proportion of these DiI-labled axons may be noradrenergic, serotoninergic or cholinergic due to the technical difficulty in combination of immunofluorescent staining and DiI labeling.

To see if there were functional experimental consequences corresponding to our anatomical data demonstrating that different slicing angles have different effects on the preservation of hippocampal pathway fibers, we compared fEPSPs evoked by stimulating PP and MF afferent pathways in HEC and coronal slices. As expected, we successfully recorded MF fEPSPs from sl in CA3 in response to stimulation of gcl in HEC slices. Consistent with the two criteria set in previous studies (Castillo et al., 1994; Urban and Barrionuevo, 1996), these MF fEPSPs had a downward signal with a duration longer than 4 ms and an upward signal would appear when the recording electrode was moved into sr with the stimulating electrode still in gcl. Moreover, the evoked MF fEPSPs could be blocked by DCG-IV, a selective MF-CA3 transmission blocker (Uchigashima et al., 2007). In contrast, we were unable to evoke MF fEPSPs from coronal slices, confirming that MFs had been severely truncated in coronal slices.

When applying electrical stimulation to sl-m of CA1 in HEC slices, we could reliably evoke fEPSPs that exhibited both glutamatergic and GABAergic components (Colbert and Levy, 1992). These fEPSPs could be sequentially blocked by bath application of APV/CNQX and bicuculline methiodide, as previously demonstrated (Colbert and Levy, 1992; Empson and Heinemann, 1995; Ito and Schuman, 2007). A large fiber volley could be always detected before the fEPSPs even when both electrodes (stimulation and recording) were situated as far as 1200 μm apart (Figure 9, Waveform 1), suggesting the presence of functional, well-preserved and long reaching fibers in HEC slices. This fiber volley increased in size when in the presence of the glutamatergic and GABAergic antagonists were present in the superfusing aCSF, as previously shown by Colbert and Levy (1992; Figure 6).

Although both DG and CA1 are major targets of entorhinal-hippocampal projection fibers, previous electrophysiological studies have to a large part focused entorhinal afferent input to the DG (Witgen et al., 2005; Cole et al., 2010). The present study is one of the first to focus PP stimulation experiments on CA1, mainly in accordance with our DiI tracing data demonstrating a prominent difference between HEC and coronal slices in how well the PP projection to CA1 is physically preserved.

### Technical Considerations

The Golgi silver impregnation method, a powerful classical technique to reveal neuronal connectivity, was used by Ramon y Cajal (1911) to systematically investigate fiber circuitry in the adult and developing hippocampus. His work entitled “Texture of the Nervous System of Man and the Vertebrates” (1911) has been consulted as a guideline for hippocampal anatomists and physiologists alike for more than a century. In the present study, we re-examined a portion of this classic work for the following reasons: (1) we wanted to evaluate how well these fibers were preserved in brain slices prepared using different slicing angles, since different slices are routinely used in physiological recording (HEC slices: Gu and Yakel, 2017; coronal: Haselmann et al., 2015; transverse: Tominaga and Tominaga, 2016); and (2) tract tracing with DiI is more efficient than Golgi staining and there is less uncertainty regarding which fibers are stained (Ogata-Iwao et al., 2011), since DiI labels all fibers while Golgi staining labels an unknown subset of fibers (Spacek, 1989). In addition, since DiI was applied to fixed whole brains and/or slices under direct vision, we could confine the tracer within the cells of origin of a desired specific pathway in the various brain slices.

To label PP fibers, we injected DiI directly into EC which was then transported in the anterograde direction along long-range projecting fibers (Ogata-Iwao et al., 2011). During the analysis of anterograde labeling experiments it is critical to exclude retrograde labeling of processes since DiI can be used for retrograde tracing (Xiong et al., 2007) as well. Although EC projects to all regions of the hippocampus, CA1 and the subiculum are the sole hippocampal origin of reciprocal projections returning to EC (Amaral and Lavenex, 2007). It has been clear that these reciprocal hippocampal-entorhinal fibers travel through the alveus (Finch and Babb, 1981) before terminating in deep layers (V and VI) of EC (Amaral and Lavenex, 2007; Witter, 2012). We made every effort to avoid DiI diffusion to EC layers V and VI by inserting the glass pipette no deeper than 300 μm from the pia. As a result, our DiI injections should target layer II/III (see van Groen et al., 2003 for a review of these connections). By restricting our injections to EC layer II/III we could label PP fibers projecting to CA1 without the complication of also labeling the dendritic arbors of CA1 pyramidal neurons. It is unclear, however, if any of DiI-labeled fibers we observed in CA1 were derived from long-range projecting GABAergic interneurons residing in EC (Germroth et al., 1989; Melzer et al., 2012) but these inhibitory fibers may be limited in number when compared to the enormous glutamatergic PP fibers (Melzer et al., 2012).

### Species Difference in Perforant Pathway

It has been documented that the DG and CA3 receive PP innervation from stellate cells in layer II of EC, whereas those targeting area CA1 are derived from pyramidal cells in layer III. This is a widely accepted doctrine based on previous studies in the rat, cat and monkey (reviewed by Amaral and Witter, 2000; Amaral and Lavenex, 2007; Witter, 2007). We, by contrast, found identical EC projections to both CA1 and CA3. Our results in mice are consistent with those of van Groen et al. (2003) who performed both antero- and retrograde tracing in the mouse and showed that mouse layer II stellate cells project to the DG alone but layer III pyramidal cells innervate CA1 to CA3. Together with other unique features of PP pathway in the mouse (van Groen et al., 2003) compared to other animals (rat: Dolorfo and Amaral, 1998; Witter et al., 1988; cat: Witter and Groenewegen, 1984; monkey: Witter and Amaral, 1991), our findings emphasize the importance of taking species differences into account (van Groen et al., 2002, 2003; Witter, 2012). That having been said, the theory of a species difference in the PP remains controversial, since the effects of different slicing angles could not be excluded, as pointed out by Witter (2012). In the present study we clearly show single PP fibers do indeed extend from CA1 to CA3 (Figure 2B, *arrow*) and provide robust evidence suggesting a common origin for PP innervation to CA1 and CA3 in the mouse.

### Different Pathway Fibers May Be Well-Preserved in sl-m in Different Slices

Heinemann and colleagues (Walther et al., 1986; Stanton et al., 1987; Jones and Heinemann, 1988) have shown that epileptiform activity could be induced in DG and CA1/CA3 in HEC slices with EC attached. These epileptiform activities disappeared when the hippocampus was isolated from EC. The authors thus suggested that EC served to generate epileptiform activity in the hippocampus (Jones and Heinemann, 1988). It has been proposed that the integrity of fiber connections between EC and hippocampus may be better preserved in HEC slices (Jones and Heinemann, 1988; Rezai et al., 2013) based on electrophysiological recordings. Here we provide anatomical evidence for this hypothesis by showing DiI-labeling of intact PP fibers in HEC slices, but never in coronal slices. The strength and reliability of the fEPSP recording in sl-m from HEC slices provided further evidence for preservation of this pathway.

It is surprising that fEPSPs could also be evoked in sl-m from coronal slices, given how truncated the PP fibers were in sl-m from coronal slices. Although large diameter PP fibers had been severely truncated in coronal slices, it needs to be ruled out if there were still enough continuous bouton-bearing collaterals of PP fibers in sl-m to generate a fEPSP. There may also be gap junctions between PP fibers that would allow groups of truncated fibers to conduct continuously. Considering that temporoammonic alvear pathway fibers could be clearly traced into sl-m only in coronal slices, we propose that these fibers may be at least partially responsible for the evoked fEPSPs in coronal slices. Upon reaching sl-m, the temporoammonic alvear pathway fibers branch and run parallel to sl-m. The terminal, laminae crossing intra-hippocampal portion of these fibers (EC-alveus to sl-m) was only visible after fixed whole brain injections and coronal sectioning, indicating that their laminae crossing processes are at least partially contained in a coronal slice, but that their extra-hippocampal portion does not travel continuously from EC to the alveus in the coronal plane. In other words, the temporoammonic alvear fibers in a coronal slice are still continuous within sl-m, and potentially capable of contributing to the sl-m evoked fEPSPs. As a result, we suggest that different pathway fibers might be responsible for the fEPSPs we observed when stimulating sl-m in these two different types of slices: PP fibers in HEC slices, but temporoammonic alvear pathway fibers in coronal slices. To test this speculation, optogenetic methods might be able to selectively manipulate PP or temporoammonic alvear pathway fibers. The optogenetic approach can be achieved by injecting virus containing the DNA sequence for an opsin behind an excitatory cell promoter into EC, and then illuminating the alveus-oriens of CA1 (for targeting alvear pathway) at its median to caudal levels or the boundary between the subiculum and CA1 (for PP). Due to the intermingling of hippocampo-entorhinal projections (Amaral and Lavenex, 2007) and pyramidal cell apical dendrites, temporoammonic alvear pathway fibers may be extremely hard to target using extracellular stimulation alone. In future studies, optogenetic approaches may be useful to uncover if any of other afferent fibers, especially those from the nucleus reuniens to CA1 (Dolleman-Van der Weel et al., 1997; Vertes, 2015) might contribute to the evoked fEPSP in sl-m using the present protocol. For instance, the reuniens inputs could be silenced and then the sl-m could be stimulated with an electrode.

## Conclusion

In summary, the present study suggests that HEC slices may be the most suitable slice preparation for studies in which all three of the major pathways must be preserved—PP, MFs and SCs. Consistent with this notion, phenomena likely to depend on the preservation of inter-regional communication have indeed been observed in HEC slices. Cappaert et al. (2009), for instance, have recorded theta wave propagation in HEC slices from EC to DG, and onward to CA3 and CA1. Similarly, Stepan et al. (2012) used HEC slices to demonstrate that evoked theta-frequency input from EC to DG effectively resulted in EPSPs in CA3 pyramidal neurons as well as LTP in CA1. If preservation of all three pathways is not needed for a particular study, then non-HEC slice may be appropriate. Although contradictory reports exist (Cai et al., 2013; Rezai et al., 2013), transverse slices may be adopted for physiological studies focusing on MFs and SCs, and coronal slices would be appropriate when the SCs or temporoammonic alvear pathway fibers are the area of interest.

## Author Contributions

GX designed the study, collected, analyzed and interpreted data, edited the manuscript. HM collected data, revised the manuscript. BNJ interpreted data, edited the manuscript. ASC designed the study, edited the manuscript.

## Conflict of Interest Statement

The authors declare that the research was conducted in the absence of any commercial or financial relationships that could be construed as a potential conflict of interest.

## Abbreviations

alv: Alveus
CA1, CA3: Area 1 or 3 of Cornu Ammonis
DG: Dentate gyrus (of the hippocampus)
EC: Entorhinal cortex
gcl: Granule cell layer
HEC (slice): Hippocampus-entorhinal cortex (slice)
MF: Mossy fiber
ml: Molecular layer
pcl: Pyramidal cell layer
PP: Perforant path
SC: Schaffer collateral
sl: Stratum lucidum
sl-m: Stratum lacunosum-moleculare
sr: Stratum radiatum.
